# Adherence to national malaria treatment guidelines in private drug outlets: a cross-sectional survey in the malaria-endemic Kisumu County, Kenya

**DOI:** 10.1186/s12936-023-04744-7

**Published:** 2023-10-11

**Authors:** Fredrick Ouma Odhiambo, Wendy P. O’Meara, Ahmed Abade, Maurice Owiny, Fredrick Odhiambo, Elvis O. Oyugi

**Affiliations:** 1 Kisumu County Department of Health, Kisumu, Kenya; 2 School of Public Health, Moi University, Eldoret, Kenya; 3 Duke Global Health Institute, Durham, NC USA; 4 Field Epidemiology and Laboratory Training Programme, Nairobi, Kenya; 5 National Malaria Control Programme, Nairobi, Kenya

**Keywords:** Kenya, Malaria, Guideline adherence, Private sector

## Abstract

**Background:**

Malaria prevalence in Kenya is 6%, with a three-fold higher prevalence in western Kenya. Adherence to malaria treatment guidelines improves care for suspected malaria cases and can reduce unnecessary anti-malarial use. Data on adherence to guidelines in retail drug outlets (DOs) is limited, yet approximately 50% of people with fever access treatment first in these outlets. This study assessed adherence to the national malaria treatment guidelines among DOs in a high transmission area of Western Kenya.

**Methods:**

In a cross-sectional survey of DOs in Kisumu Central and Seme sub-counties in 2021, DO staff were interviewed using structured questionnaires to assess outlet characteristics (location, testing services), staff demographics (age, sex, training), and health system context (supervision, inspection). Mystery shoppers (research assistants disguised as clients) observed malaria management practices and recorded observations on a standardized tool. Adherence was defined as dispensing artemether-lumefantrine (AL) to patients with a confirmed positive test, accompanied by appropriate medication counseling. Logistic regression was used to test for association between adherence to guidelines and DO-related factors.

**Results:**

None of the 70 DOs assessed had a copy of the guidelines, and 60 (85.7%) were in an urban setting. Staff adhered to the guidelines in 14 (20%) outlets. The odds of adherence were higher among staff who had a bachelor’s degree {odds ratio (OR) 6.0, 95% confidence interval (95% CI) 1.66–21.74}, those trained on malaria rapid diagnostic test (RDT) {OR 4.4, 95% CI 1.29–15.04}, and those who asked about patient’s symptoms {OR 3.6, 95% CI 1.08–12.25}. DOs that had higher odds of adherence included those with functional thermometers {OR 5.3, 95% CI 1.46–19.14}, those recently inspected (within three months) by Pharmacy and Poisons Board (PPB) {OR 9.4, 95% CI 2.55–34.67}, and those with all basic infrastructure {OR 3.9, 95% CI 1.01–15.00}. On logistic regression analysis, recent PPB inspection {adjusted OR (AOR) 4.6, 95% CI 1.03–20.77} and malaria RDT-trained staff (aOR 4.5, 95% CI 1.02–19.84) were independently associated with adherence.

**Conclusion:**

Most outlets didn’t adhere to malaria guidelines. Regular interaction with regulatory bodies could improve adherence. Ministry of Health should enhance private sector engagement and train DOs on RDT use.

## Background

Malaria is a preventable and curable life-threatening disease caused by *Plasmodium* parasites transmitted to people through infected female *Anopheles* mosquitoes. There were 229 million cases and over 409,000 deaths from malaria globally in 2019 [1]. Africa bears the most significant malaria burden, with 215 million malaria cases and 384,000 malaria deaths in 2019. Kenya, one of the fifteen high-burden malaria countries in sub-Saharan Africa, reports that over 18% of outpatient consultations are related to malaria [2, 3]. Approximately 20% of all health facility admissions are due to malaria [4]. The prevalence of malaria was 6% in 2020 [5], with *Plasmodium falciparum* accounting for 92% of all cases [3].

Private drug outlets are among the sources of advice on malaria treatment and medicines for 18% of the residents of the endemic lake region. About 40% of the residents of urban areas seek advice and treatment from private health facilities and shops [5]. Other sources of advice on malaria treatment include faith-based health facilities, government-owned public health facilities, private hospitals and medical centers, and traditional healers. Less than 5% of suspected malaria cases visiting private drug outlets are tested for malaria [5]. Those who are not tested receive presumptive treatment. The involvement of private sector drug outlets in malaria testing using RDTs is projected to improve adherence to malaria treatment guidelines [6]. In recent years, the capacity building of healthcare providers has been extended to community health workers and private health facilities [7]. However, there is a capacity gap in private drug outlets, which serve as primary healthcare institutions and from which many people purchase malaria medicines conveniently [8]. The knowledge of malaria treatment guidelines is higher among health facility providers than drug outlet dispensers [9]. In a day, drug outlets may serve dozens of suspected cases [10].

Outlets that stock RDT or conduct malaria tests using RDT purchase the kits from the private market since the government only supplies the kits to public and faith-based health facilities.

The Kenya Malaria Strategy outlines six ambitious goals to combat malaria. The second is to manage 100 percent of suspected malaria cases according to the Kenya National Malaria Treatment guidelines by 2023 [3]. The 6th edition of the treatment guidelines recommends testing all patients with uncomplicated malaria symptoms using microscopy or rapid diagnostic tests (RDTs) as confirmatory tests to detect the presence of malaria parasites. Only those who test positive for malaria should receive treatment using artemether-lumefantrine (AL, first-line treatment) or, in the absence of AL, dihydroartemisinin-piperaquine (DHAP, second-line treatment), at the correct weight-based dosage (Appendix 7) [8]. However, pregnant women with confirmed uncomplicated malaria in the first trimester should receive quinine tablets unless this is unavailable. Proper instructions should be given on administering the medicines, including preparing dispersible tablets for children, dosing intervals, and the need to complete the dose over three days, even if the patient already feels better. If vomiting occurs within 30 min of administration, a repeat dose should be taken. If vomiting persists or the condition deteriorates, the patient should be reviewed in a health facility [8, 11].

The availability of subsidized ACT has led to increased accessibility of malaria medicines, which might lead to misuse of the commodities [12]. About half of the patients who test negative for malaria still receive ACT presumptively [13], a situation that may contribute to the development of anti-malarial resistance, which poses a severe threat to human health [14]. However, legal constraints hinder the scaling up of RDT use in the private sector. For instance, a court ruling in April 2019 prohibited non-skilled laboratory staff from carrying out tests like malaria RDT because it violates various constitutional provisions [15]. This court ruling resulted from a case filed by the Kenya Medical Laboratory Technologists and Technicians Board, the regulatory body for laboratory officers. They argued that allowing non-laboratory staff to conduct diagnostic tests violates the constitutional clause, which states that all Kenyans are entitled to the highest attainable healthcare standards. The context of the lawsuit was that community health workers (with no medical qualification) were conducting malaria RDT testing at the community level [15]. The resulting ruling meant that pharmacists, pharmaceutical technologists, nurses, and clinicians should not be allowed to perform diagnostic tests. However, in actual practice at lower-level health facilities, there needs to be more laboratory officers; hence, the available staff conduct these tests. This has generally been allowed by the Ministry of Health under the guidelines on task sharing to improve access to healthcare services. The court ruling implies that individuals working as drug dispensers in the private informal sector are barred from conducting malaria diagnostic tests. Despite this, some private drug retail outlets continue to perform malaria RDT or dispense anti-malarials over the counter. Given the high use of the private retail sector in these malaria-endemic communities, this study assessed the level of adherence to the national malaria treatment guidelines and the factors associated with adherence thereto.

## Methods

### Study design and setting

Kisumu County is part of the endemic lake region, whose malaria prevalence in children under 14 years was 26.7% in 2015 [16] and 19% in 2020 [5]. Between 2018 and 2019, a total of 1804 health facilities in the lake endemic counties reported testing a total of 12.8 million outpatients, with about 6 million of the tests turning positive (test positivity rate 46.7%, mean 309 cases in primary health facilities) in the 24 months [17, 18]. A survey conducted in schools in Kisumu County revealed that each person experiences at least 31 infective bites annually [19].

**Fig. 1: The conceptual framework for adherence to treatment guidelines in private drug outlets in Kisumu, Kenya:**
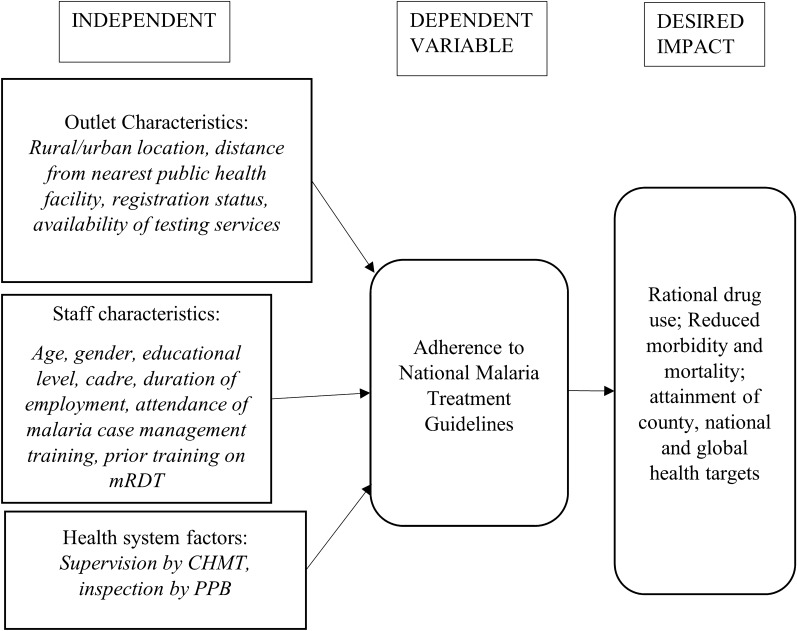
.

The study was a cross-sectional survey. Private drug outlets were sampled from Kisumu Central and Seme sub-counties, and an assessment was carried out on the adherence to malaria treatment guidelines from May to December 2021. Knowledge and practices on malaria case management were evaluated using a developed tool with established thresholds based on specific indicators (Fig. 1).

**Fig. 2: Map of Kisumu County, Kenya, showing the study sites:**
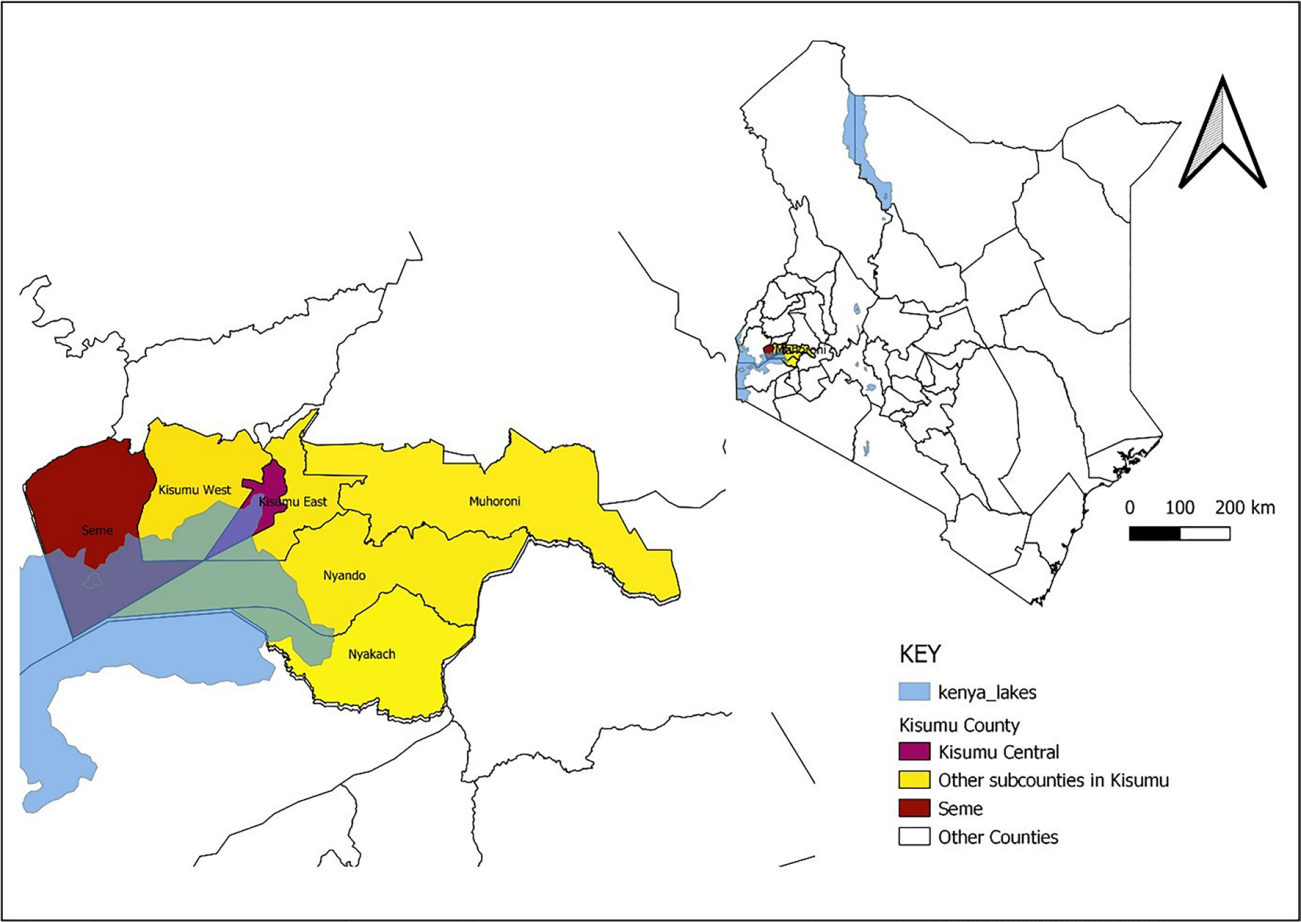
.

Kisumu County has over 120 government health facilities, 17 faith-based health facilities, 17 non-governmental organization facilities, and over 50 private health facilities [20, 21] is divided into seven administrative units called sub-counties (Fig. 2). Seme sub-county has the county's highest population-weighted mean malaria test positivity rate (about 60%), while Kisumu Central sub-county has the lowest at around 35% [10]. However, Kisumu Central hosts the more significant part of the cosmopolitan city of Kisumu and most of the region’s most extensive public and private health facilities [20]. In 2020, there were over 1.8 million and 110,000 new outpatient department attendances and admissions, respectively. Of the 637,298 suspected malaria cases, 336,302 (53%) were confirmed, resulting in 1373 deaths (case fatality rate 0.41%). Children under five years comprised over a quarter of the 3126 confirmed severe malaria admissions, with 15 fatalities reported [22]. The number of cases is usually highest around July [10].

Insecticide-treated mosquito net (ITN) coverage is 70%, while Intermittent Preventive Therapy in Pregnancy (IPTp) coverage is 35.3%; the mean number of doses of IPTp received by pregnant women in Kisumu County is 3.8, with over 40% receiving at least five doses [23]. A challenge with data on malaria is that reporting rates among private health facilities are very low [24]. The county experiences bimodal rainfall distribution, with the long rains falling between March and June while short rains fall between September and December. Temperature varies with altitude and season, ranging between a minimum of 9 °C and a maximum of 35 °C [21].

### Study population and sampling technique

The study population consisted of staff in stand-alone private drug outlets (registered and unregistered) in Kisumu Central sub-county and Seme sub-county. A comprehensive list of potentially eligible drug outlets is not available in the national or county databases, so the identification of outlets for inclusion was carried out through an outlet census. The sub-county pharmacists were contacted for clues on drug outlets' locations. A team consisting of the researcher and three research assistants was then deployed to both sub-counties to locate the mentioned outlets. Each outlet’s name and GPS coordinates were taken, and the available staff asked where the neighbouring outlets were. After the location of all the mentioned outlets, the team conducted a mop-up exercise by identifying all the private drug outlets by ward and estate/village. A total of 85 outlets met the criteria for selection. Due to the relatively small number of outlets, all of them were considered for inclusion in the study without sampling. However, only 70 outlets consented to participate. With the outlets included, it was possible to measure the prevalence of adherence to treatment guidelines to within five percentage points of the actual value with a confidence of 95%.

### Inclusion and exclusion criteria

#### Inclusion criteria

Private drug outlets that diagnose malaria and sell anti-malarial medicines on retail terms.

Private drug outlets that fill prescriptions for malaria treatment from other health facilities.

#### Exclusion criteria

Private drug outlets owned and operated jointly with medical laboratories or clinics.

### Data collection methods and data quality control

Standard indicators were constructed according to definitions used by the Ministry of Health as captured in the Kenya Malaria Monitoring and Evaluation Plan. Under the KMS’s second objective (to manage 100% of suspected malaria cases according to the Kenya malaria treatment guidelines by 2023), some of the indicators used are the proportion of suspected malaria cases tested with RDT and microscopy and the proportion of suspected malaria cases managed according to the guidelines [3]. Adherence to the guidelines was assessed by evaluating testing or asking to see laboratory results, selecting appropriate treatment, and patient counseling.

#### Outlet staff interview

Study data collectors introduced themselves to the outlet staff and sought an audience with the superintendent or person dispensing drugs. Permission to collect data was sought from the outlet’s owner/manager/director as applicable in case of outlets where the staff did not have the authority to allow access to such information. In such a case, the interviewer provided the information contained in the consent form to the owner/manager/director to ensure clarity and avoid suspicion. After obtaining consent for both the interview and the mystery client, the interviewer started administering the first question of the survey questionnaire but was interrupted by the entrance of the mystery client (study staff), as described below.

After the exit of the mystery client, the interviewer proceeded with the interview, collecting information on the outlet’s drug dispensers’ demographics, pre-service training, access to guidelines, retrospective exposure to in-service training and supervision, availability and storage of various anti-malarial drugs, the availability of malaria diagnostic services, and the availability of appropriate basic equipment. Appropriateness of documentation was assessed by verifying the availability of ledger books or other inventory records (physical or electronic), copies of delivery notes and invoices, and pharmacovigilance reporting forms (pink and yellow forms) or proof of previously submitted reports (paper or electronic). The outlet assessment was conducted using both direct observation and interview.

#### Mystery client technique

To reduce suspicion and minimize information bias (Hawthorne effect), the study staff pretending to be a client (mystery client) presented immediately after consent had been obtained by the drug outlet staff interviewer, posing as a client (mystery client), and the interviewer excused themself and moved away to allow the mystery client to consult and be attended to privately. The mystery client helped check the usual practice at the drug outlet in terms of diagnosis, choice of medicine, and dispensing practices using a standard case scenario. The standard mystery client script described a 4-year-old child at home with malaria. If asked for the symptoms, they mentioned hotness of body, refusal to feed, and generally sick-looking over the previous two days. The mystery client had a copy of positive malaria microscopy results indicating malaria parasites (MPS + +) seen. This was only presented if asked for by the staff. Interviewer bias was minimized by advanced training of the research assistants on the case scenario and the indicators to look out for.

After the encounter, the information was recorded on a standard tool designed to answer critical dispensing and counseling processes observed during the visit.

### Variables of the study

Adherence to malaria treatment guidelines was defined as dispensing the right malaria medicines only to a client with evidence of a positive malaria test result from microscopy or RDT, accompanied by the provision of appropriate instructions on the use of the medicines. This outcome was assessed based on the mystery shopper tool.

Secondary outcomes included appropriate drug outlet infrastructure, adequate stocking levels, staff skills in malaria case management, and good record-keeping practices. These outcomes were assessed based on the drug outlet staff tool.

### Data processing and analysis

Continuous variables were summarized using central tendencies and dispersion measures, while categorical variables were summarized using frequencies and proportions. The Chi-square test was used to assess the association between dependent (adherence to treatment guidelines) and independent variables (provider socio-demographics, other provider characteristics, outlet characteristics, and health system factors) calculated for the adherence to treatment guidelines. Any variable with a p-value of less than 0.05 at the bivariate level was subjected to multivariable logistic regression. All variables with a p-value less than 0.05 at the multivariate level were regarded as independently associated with adherence to malaria treatment guidelines. Analysis was done using Microsoft Excel and EpiInfo software.

## Results

### Socio-demographic characteristics of staff in private drug outlets in Kisumu County

#### Drug outlets

A total of 70 drug outlets were assessed during the study period, with 60 (85.7%) being in an urban setting (Kisumu Central sub-county) and 10 being in a rural setting (Seme sub-county). All (100%) were reportedly registered and displaying a Pharmacy and Poisons Board registration license. Of the assessed outlets, 17.1% (12) had all four aspects of infrastructure (electricity 98.6%, safe drinking water 51.4%, functional thermometer 40.0%, and weighing scale 31.4%). Stock out of AL had been experienced within the previous three months in 4.3% (3) outlets, while RDT had recently been stocked out in 97.1% (68) outlets. Reasons for commodity stock out included lack of a suitable supplier and delayed delivery of ordered commodities. Malaria diagnostic services (using RDT) were available in 50.0% (35) outlets, suggesting that many shops sold RDTs but did not perform them at the outlet.

Of the assessed outlets, 70.0% (49) had an average workload of fewer than 50 clients daily. The average number of suspected malaria cases attended to in the outlets was 7 (SD 3.2), ranging from 1 to 20 patients. None of the outlets had a copy of the national malaria treatment guidelines. Proper documentation, defined as the availability of ledger books or other inventory records (physical or electronic), copies of delivery notes/invoices, and pharmacovigilance reporting forms (pink and yellow forms) or proof of previously submitted reports (paper or electronic), was verified in 65.7% (46) of the outlets. One outlet had ledger books, 62.9% (44) had copies of receipts and invoices, 27.1% (19) had copies of delivery notes, 5.7% (4) had pink form for reporting poor quality medicinal products, and 1.4% (1) had the yellow suspected adverse drug reaction reporting forms (Table 1).

**Table 1:** Characteristics of private drug outlets in Kisumu County, 2021

Variable	Frequency	Proportion
Location		
Urban (Kisumu Central sub-county)	60	85.7
Rural (Seme sub-county)	10	14.3
Basic infrastructure		
Electricity	69	98.6
Safe drinking water	36	51.4
Thermometer	28	40.0
Weighing scale	22	31.4
All basic infrastructure	12	17.1
AL Stock-out		
No	67	95.7
Yes	3	4.3
Diagnostic services available		
Yes	35	50.0
No	35	50.0
Overall daily workload		
High (100 + clients)	6	8.6
Moderate (50–99)	15	21.4
Low (< 50 clients)	49	70.0
Malaria workload		
≤ 5 suspected cases	26	37.1
> 5 suspected cases	44	69.2
Good documentation	46	65.7
Recently supervised/inspected	14	20.0
Recently supervised by CHMT	6	8.6
Recently inspected by PPB	11	15.7
Staff age group		
30–39 years	30	42.9
20–29 years	27	38.6
40–49 years	8	11.4
50–59 years	5	7.1
Staff highest education level		
Diploma	50	71.4
Bachelor’s degree	14	20.0
Secondary school certificate	5	7.2
Post-graduate diploma	1	1.4
Malaria case management training		
Recent (after 2016)	34	48.6
2014–2016	17	24.3
None	19	27.1

In the preceding three months, 14 (20.0%) outlets had received a supervisory or inspectorate visit, including 11 (15.7%) being inspected by the Pharmacy and Poisons Board (PPB) and 6 (8.6%) being supervised by the County Health Management Team (CHMT) (Table 1).

#### Outlet staff

**Fig. 3: Age groups of staff in private drug outlets in Kisumu County, Kenya, on day of visit, 2021:**
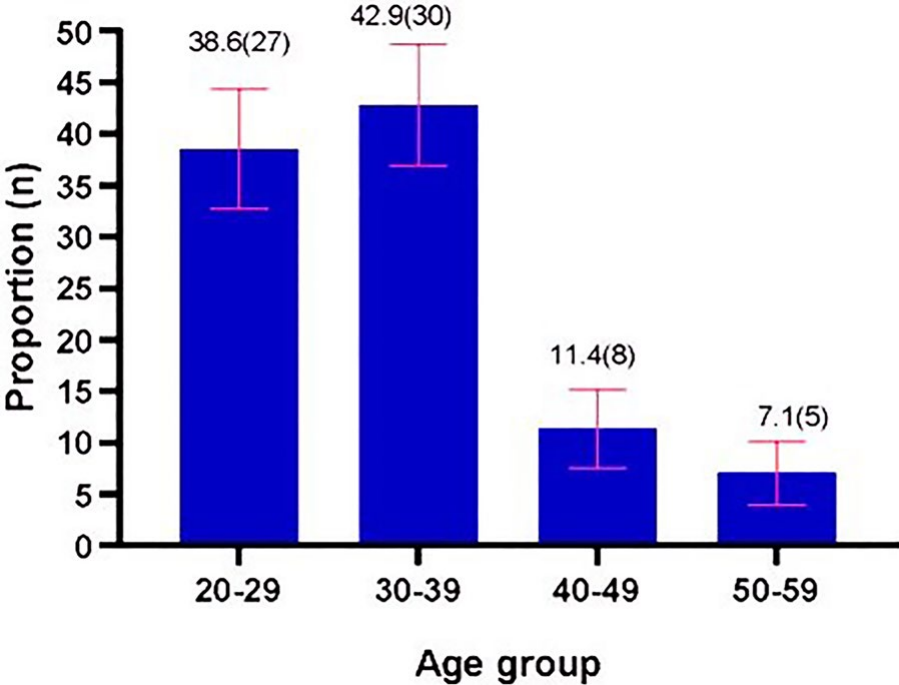
.

On the assessment days, 71.4% (50) of the drug outlets had pharmacists or pharmaceutical technologists dispensing. Males constituted 50.0% (35) of the drug outlet staff interviewed. The age group of 30–39 years formed 42.9% (30) of the staff (Fig. 3). Of the staff, 71.4% (50) had a diploma as the highest level of education. Staff who had ever been trained on RDT use constituted 32.9% (23), while those who had recently been trained in malaria case management (after 2016) constituted 48.6% (34) of the staff (Table 1).

#### Adherence to national treatment guidelines for uncomplicated malaria

A total of 70 mystery client visits were conducted. In 16 (22.9%) instances, the staff asked to see documentation of a malaria test result. Correct first-line malaria treatment was offered to 14 (87.5%) patients for whom the diagnostic report was observed. Overall, 20.0% (14) of private drug outlets adhered fully to the Kenya National Malaria Treatment Guidelines.

23 (32.9%) clients were asked about the symptoms experienced. Seven (10.0%) mystery shoppers were offered a malaria test or referred for testing. A question on diagnostic testing was asked in all cases where antimalarial medication was dispensed. The first-line anti-malarial (AL) was recommended in 92.9% (65) of the outlets (regardless of test status), whereas 74.3% (52) outlets offered complete instructions on the use of the medicine (Tables 2).

**Table 2:** Bivariate analysis of outlet and system factors in Kisumu private drug outlets, 2021

Variable	Adhered to guidelines		Prevalence ratio (95% CI^a^)	P value
	Yes	No		
Recently supervised/inspected				
No	6	42	Reference	–
Yes	8	14	4.0 (1.18–13.54)	0.0205
Recently inspected by PPB^b^				
No	5	47	Reference	–
Yes	9	9	9.4 (2.55–34.67)	0.0002
Recently supervised by CHMT^c^				
No	9	50	Reference	–
Yes	5	6	4.6 (1.16–18.45)	0.0215
Have basic infrastructure				
No	9	49	Reference	–
Yes	5	7	3.9 (1.01–15.00)	0.0393
Have functional thermometer				
No	4	38	Reference	–
Yes	10	18	5.3 (1.46–19.14)	0.0130
Have functional weighing scale				
No	7	41	Reference	-
Yes	7	15	2.7 (0.82–9.10)	0.0942
Offer diagnostic services				
No	4	31	Reference	–
Yes	10	25	3.1 (0.87–11.08)	0.1334
Location				
Rural	1	9	Reference	–
Urban	13	47	2.5 (0.29–21.49)	0.6742
Overall daily workload				
Moderate to high (at least 50 clients)	4	17	Reference	–
Low (less than 50 clients)	10	39	1.1 (0.30–3.97)	1.0000
Daily malaria workload				
Low (less than 10 suspected cases)	9	43	Reference	–
High (at least 10 suspected cases)	5	13	1.8 (0.52 – 6.46)	0.3385
Proper documentation				
No	3	21	Reference	–
Yes	11	35	2.2 (0.55–8.80)	0.3518
Have safe drinking water				
No	6	28	Reference	–
Yes	8	28	1.3 (0.41–4.34)	0.6324

^a^Confidence interval

^b^Pharmacy and poisons board

^c^County health management team

**Table 3:** Bivariate analysis of staff factors in Kisumu County private drug outlets, 2021

Variable	Adhered to guidelines		Prevalence odds ratio (95% CI^a^)	P value
	Yes	No		
Have a bachelor’s degree				
No	7	48	Reference	–
Yes	7	8	6.0 (1.66–21.74)	0.0036
Staff asked about the symptoms				
No	6	41	Reference	–
Yes	8	15	3.6 (1.08–12.25)	0.0305
Staff trained on mRDT^b^ use				
No	6	43	Reference	–
Yes	8	13	4.4 (1.29–15.04)	0.0132
Pharmacy-trained staff				
No	1	16	Reference	–
Yes	13	40	5.2 (0.63–43.11)	0.1620
Sex of staff				
Female	5	31	Reference	–
Male	9	25	2.2 (0.66–7.51)	0.1884
Staff ever trained in malaria case management				
No	3	16	Reference	–
Yes	11	40	1.5 (0.36–5.96)	0.7437
Recent malaria case management training (after 2016)				
No	6	30	Reference	–
Yes	8	26	1.5 (0.47–5.01)	0.4731
Age of staff				
At least 40 years	1	11	Reference	–
Under 40 years	13	45	3.2 (0.37–26.96)	0.4362

^a^Confidence interval

^b^Malaria rapid diagnostic test

#### Factors associated with adherence to the national malaria treatment guidelines

##### Bivariate analysis

The odds of adherence to malaria treatment guidelines were 3.9 times higher among the outlets with all four basic infrastructures than those without one or more (p = 0.0393). Those with functional thermometers had 5.3 times higher odds of adhering to the guidelines than those who did not have (p = 0.0130). The odds of adherence were four times higher among outlets that had received a supervisory or inspectorate visit within the preceding 3 months than those who had not (p = 0.0205), with the odds being 9.4 times higher among those that had been inspected by the Pharmacy and Poisons Board (PPB) than those that had not been inspected by PPB (p = 0.0002).

The odds of adherence to the guidelines were 2.2 times higher in male staff than in females (p = 0.1884). The odds of staff with bachelor’s degrees adhering to guidelines were 6 times higher than those without (p = 0.0036). Those who had been trained on RDT use had 4.4 times higher odds of adherence to the guidelines than those who had not been trained (p = 0.0132). The odds of adherence were 3.6 times higher among staff who asked about other signs and symptoms of the patient (p = 0.0305) and 3.8 times higher among staff who asked if the patient had been tested (p = 0.0271) than those who did not ask (Table 3).

##### Logistic regression analysis

A multivariable logistic regression of crucial variables was used to identify factors independently associated with adherence to treatment guidelines. The multivariable logistic regression included variables with a p-value of less than 0.05. However, co-linear variables such as recent supervision/inspection and recent supervision by CHMT were left out since private drug outlets were not ordinarily expected to be supervised by CHMT but by PPB.

Among the factors included in the model, only the following were significantly associated with the outcome: Staff trained on RDT use (p = 0.0471, 95% CI 1.02–19.84) and outlet recently inspected by PPB within the preceding three months (p = 0.0459, 95% CI 1.03–20.77). Other factors subjected to multivariable analysis but not statistically significant included staff asking about patients’ symptoms and staff having a bachelor’s degree (Table 4).

**Table 4:** Logistic regression analysis for factors associated with adherence to malaria guidelines, private drug outlets, Kisumu

Variable	Adjusted odds ratio	95% Confidence interval	P-value
Staff trained on RDT			
No	Reference	–	–
Yes	4.5	1.02–19.84	0.0471
Outlet recently inspected by PPB			
No	Reference	–	–
Yes	4.6	1.03–20.77	0.0459
Staff has a bachelor’s degree			
No	Reference	–	–
Yes	3.6	0.74–17.80	0.1115
Staff asked about symptoms			
No	Reference	–	–
Yes	2.5	0.57–11.07	0.2267

## Discussion

Kisumu County has one of the highest malaria prevalence in the country, two and a half-fold higher than the general country-wide prevalence. Approximately three-quarters of the outlets visited had a relatively low malaria workload, attending to less than ten suspected malaria cases daily. This contradicts the findings in a survey conducted in the same region, which indicated that drug outlets serve between 10 and 250 suspected cases daily [25]. This could be due to reduced malaria prevalence, as noted by the most recent malaria indicator survey that showed reduced malaria prevalence in the region from 27 to 19% [5]. However, this study found that the overall and malaria-specific workload were not associated with adherence to the national malaria treatment guidelines.

Investment should be made in the private sector to prevent the over-prescription of anti-malarial medicines and improve adherence to the national guidelines on case management of malaria [12]. Through the National Malaria Control Programme (NMCP), Kenya’s Ministry of Health revised and disseminated the national guidelines for the diagnosis, treatment, and prevention of malaria in 2020. This new edition of the guidelines was availed in hard copies to key county and sub-county health management teams. It was also available for circulation in soft copy and uploaded to the DNMP website (www.nmcp.or.ke). During this study, it was noted that none of the outlet staff had a copy of the guidelines. This differed significantly from a national survey conducted in September 2018 that found approximately a fifth of outlet staff had access to copies of the guidelines [17] and an earlier similar national survey in which only 15% had access to the guidelines [26]. The 5th edition (released in 2016) was in use by then. This decline in access to guidelines could indicate that the dissemination of the guidelines did not target the private sector. It could also mean that the private sector health workers needed to be sensitized on the availability of the digital version and where it could be obtained, pointing to the need for extensive stakeholder engagement during such dissemination. Since the questionnaire sought to collect data on the availability of guidelines without reference to the most recent, it was unclear if the outlets in this study had been part of the 2018 survey that had reported having some guidelines and where the previous versions were, if any. The outlet staff could have been relying on widespread messaging by the DNMP on using artemether-lumefantrine (AL) to treat malaria without seeing the need to seek additional information.

This study defines adherence to the national malaria treatment guidelines as dispensing the right medicine (AL) to a client with proof of a positive malaria test and issuing correct dosing instructions (number of tablets, dosing frequency, and duration of treatment). Only a fifth of the outlets adhered to the guidelines. This was lower than the 57.8% reported in the national private sector quality of care survey conducted in 2016 [26]. This decline in adherence could be attributed to the fact that none of the outlets was found to have a copy of the national guidelines. As noted earlier, a lack of guidelines would mean that providers rely on general social behaviour change messaging from DNMP on malaria, with a high chance of forgetting some or all the essential aspects of the messages. However, this finding was comparable to a survey in Malawi, which reported 26% adherence [27].

Most mystery clients were offered treatment without confirmation of a positive test for malaria from a laboratory or even asking that the child be taken for parasitological diagnosis. Many patients presenting to private drug outlets receive AL without a positive malaria test [12, 17, 18, 28]. This is referred to as presumptive treatment. This points to a gap in the ‘first T’ of the Ministry of Health’s Test-Treat-Track policy, which requires that all suspected malaria cases be tested (Test) and only confirmed positive patients be treated (Treat) and followed up (Track). This policy is the backbone of the DNMP’s malaria case management unit as per the second objective of the Kenya Malaria Strategy (KMS, 2019–2023) [3].

The NMCP periodically conducts training of healthcare workers on malaria case management. During such training, there are modules on malaria diagnosis, including the use of malaria RDT. Diagnosis starts with the identification of suspected malaria cases. Proper history taking, including eliciting the signs and symptoms, is necessary. This study found some positive association between staff asking for patients’ symptoms and adherence to the guidelines, though this association was not statistically significant. The proportion of outlets whose staff asked about the patient’s signs and symptoms was lower than that found in modelling done in Nigeria, Uganda, and Tanzania in 2019 [29]. This difference could be due to variations in the malaria control strategies and implementation of activities, specifically private sector involvement.

After adequately identifying a suspected case, the guidelines recommend testing them for malaria using an appropriate test—microscopy or RDT. For stand-alone drug outlets, it is more feasible to diagnose malaria using RDT, so long as the staff has been capacity-built on the use of the same. The study found that drug outlet staff who reported having been trained on the use of malaria RDT were likelier to adhere to the guidelines than those who had not been trained. This aligns with findings on staff capacity and adherence to malaria treatment guidelines in two studies [30, 31]. The NMCP has put in place several strategies to improve the capacity of healthcare workers to conduct malaria diagnostic testing, including capacity building through training, mentorship, and technical supportive supervision [32, 33]. Before a case was filed in court and an order issued barring non-laboratory personnel from conducting diagnostic tests [15], there had been increased availability of RDTs in private drug outlets in Kenya with a concomitant increased availability of testing services in outlets [34] as alluded to by other studies [25]. By 2016, about 5% of malaria tests in the country were conducted in drug outlets, mainly by RDT [35]. Several studies found a positive correlation between the availability of testing services, rational drug use, and improved health outcomes [35, 36]. Therefore, it is evident that training outlet staff and availing of testing services using RDT in drug outlets will improve adherence to malaria treatment guidelines and benefit public health in general.

Upon a positive malaria diagnosis, the patient should be treated using the appropriate medicines and provided with adequate instructions on using the medication. The case scenario used in this study portrayed a picture of a child with uncomplicated malaria. The DNMP recommends using AL to treat uncomplicated malaria [8]. In this study, most outlets offered AL to treat the case. Other studies and surveys had similar findings [17, 18, 36]. This indicates that the biggest challenge in adherence to malaria treatment guidelines is not the choice of medicine. Still, on who should get a prescription, that is, diagnosis, as noted above. This further emphasizes the need to ensure drug outlet staff can correctly perform RDT diagnostic testing of suspected malaria cases. This will also reduce the knowledge gap noted by previous studies like a cross-sectional study that employed exit interviews and the mystery client technique to assess the management of fever cases in the Kenyan coastal region [6].

The Pharmacy and Poisons Board (PPB) is the country’s statutory body regulating pharmacy practice and trade in medicines. This study that recent inspection by the PPB was one of the factors associated with adherence to the malaria treatment guidelines in private drug outlets. This appears to be contrary to findings from a mixed effects logistic regression modelling of predictors of presumptive malaria treatment in private drug outlets in a 2018 national survey, which suggested that supervisory visits only improved malaria treatment practices by 1% [17]. However, this study’s findings agree with a 2018 paper on rational drug use, which asserted that a proper regulatory framework, including monitoring of operations in a drug outlet, is critical for ensuring the appropriate use of medicines [37]. Similarly, these findings agree with an earlier national survey, which concluded that the quality of treatment in the private sector requires, among other things, proper regulation of outlets [35].

### Limitations

During the outlet census and data collection processes, the team encountered no regular retail shop that was not a pharmacy. The team was later informed that there had been an inspection exercise by PPB in the county, raising alertness amongst traders when selling to non-locals or unfamiliar faces. Had this been realized earlier, a possible solution would have been to work with relatively well-known locals who had clues on eligible general retail shops. Nevertheless, given the comprehensive nature of the data collection tools and the practicality of the Ministry of Health implementing interventions based on the findings, this study gives insight into the challenges of malaria case management in a relatively more organized and regulated sector with opportunities to collaborate and improve the situation.

This study could not assess the management of malaria in pregnancy and severe malaria since these would have posed logistic and ethical challenges in simulation or getting an actual patient. Nonetheless, uncomplicated malaria in children is more common. Therefore, this study provided valuable insight into the management of malaria in private sector outlets.

## Conclusions

None of the private drug outlets in Kisumu had a copy of the national malaria treatment guidelines. Most of the outlets did not adhere to the national malaria treatment guidelines. Adherence to the guidelines was associated with the outlet staff’s prior training on using malaria rapid diagnostic test kits and regulatory inspection of the outlet by the Pharmacy and Poisons Board within the preceding three months. The Pharmacy and Poisons Board should strengthen supervisory visits to private drug outlets to enforce all regulatory requirements. The visits should be conducted in all outlets at least every quarter.

The Ministry of Health should strengthen collaboration with the private sector by involving drug outlets in disseminating national treatment guidelines and implementing the training of drug outlet staff on malaria case management. Specifically, the ministry should focus on capacity building these staff on malaria diagnosis using rapid diagnostic test kits.

## Data Availability

The datasets used during the current study are available from the corresponding author upon reasonable request.

## Abbreviations

95% CI: 95 Percent confidence interval
ACT: Artemisinin-based combination therapy
aOR: Adjusted odds ratio
CHV: Community health volunteer
CME: Continuous medical education
DO: Drug outlet
FELTP: Field epidemiology and laboratory training program
IPTp: Intermittent preventive therapy in pregnancy
IREC: Institutional research and ethics committee
ITN: Insecticidal-treated net
KMIS: Kenya malaria indicator survey
KMS: Kenya malaria strategy
NMCP: National Malaria Control Program
PPB: Pharmacy and poisons board
RDT: Rapid diagnostic test
SDGs: Sustainable development goals
WHO: World Health Organization
